# Proteomic and Metabolomic Analyses of Vanishing White Matter Mouse Astrocytes Reveal Deregulation of ER Functions

**DOI:** 10.3389/fncel.2017.00411

**Published:** 2017-12-20

**Authors:** Lisanne E. Wisse, Renske Penning, Esther A. Zaal, Carola G. M. van Berkel, Timo J. ter Braak, Emiel Polder, Justin W. Kenney, Christopher G. Proud, Celia R. Berkers, Maarten A. F. Altelaar, Dave Speijer, Marjo S. van der Knaap, Truus E. M. Abbink

**Affiliations:** ^1^Pediatrics, VU University Medical Center, Amsterdam, Netherlands; ^2^Biomolecular Mass Spectrometry and Proteomics Group, Utrecht Institute for Pharmaceutical Sciences, Bijvoet Center for Biomolecular Research, Utrecht University, Utrecht, Netherlands; ^3^Centre for Biological Sciences, University of Southampton, Southampton, United Kingdom; ^4^Medical Biochemistry, Academic Medical Center, Amsterdam, Netherlands

**Keywords:** vanishing white matter, eIF2B, AHA, SILAC, astrocytes, metabolomics, endoplasmic reticulum

## Abstract

Vanishing white matter (VWM) is a leukodystrophy with predominantly early-childhood onset. Affected children display various neurological signs, including ataxia and spasticity, and die early. VWM patients have bi-allelic mutations in any of the five genes encoding the subunits of the eukaryotic translation factor 2B (eIF2B). eIF2B regulates protein synthesis rates under basal and cellular stress conditions. The underlying molecular mechanism of how mutations in eIF2B result in VWM is unknown. Previous studies suggest that brain white matter astrocytes are primarily affected in VWM. We hypothesized that the translation rate of certain astrocytic mRNAs is affected by the mutations, resulting in astrocytic dysfunction. Here we subjected primary astrocyte cultures of wild type (wt) and VWM (*2b5^ho^*) mice to pulsed labeling proteomics based on stable isotope labeling with amino acids in cell culture (SILAC) with an L-azidohomoalanine (AHA) pulse to select newly synthesized proteins. AHA was incorporated into newly synthesized proteins in wt and *2b5^ho^* astrocytes with similar efficiency, without affecting cell viability. We quantified proteins synthesized in astrocytes of wt and *2b5^ho^* mice. This proteomic profiling identified a total of 80 proteins that were regulated by the eIF2B mutation. We confirmed increased expression of PROS1 in *2b5^ho^* astrocytes and brain. A DAVID enrichment analysis showed that approximately 50% of the eIF2B-regulated proteins used the secretory pathway. A small-scale metabolic screen further highlighted a significant change in the metabolite 6-phospho-gluconate, indicative of an altered flux through the pentose phosphate pathway (PPP). Some of the proteins migrating through the secretory pathway undergo oxidative folding reactions in the endoplasmic reticulum (ER), which produces reactive oxygen species (ROS). The PPP produces NADPH to remove ROS. The proteomic and metabolomics data together suggest a deregulation of ER function in *2b5^ho^* mouse astrocytes.

## Introduction

Vanishing white matter (VWM) is one of the more prevalent leukodystrophies (van der Knaap et al., 1999). Patients with VWM display chronic neurological deterioration and additionally episodes of stress-provoked rapid and severe deterioration. Neuropathology of post-mortem brain shows cystic degeneration of the cerebral white matter with lack of appropriate astrogliotic scar formation, profound lack of myelin, increased numbers of oligodendrocyte precursor cells and immature astrocytes. The morphology of especially astrocytes in cerebral white matter is abnormal. They look coarse and have fewer, thicker processes than normal (van der Knaap et al., 2006; Bugiani et al., 2011). A small proportion of the oligodendrocytes look foamy (Wong et al., 2000).

Recessive mutations in the eukaryotic translation factor 2B (eIF2B) cause VWM (Leegwater et al., 2001; van der Knaap et al., 2002). eIF2B is an enzyme composed of five different subunits (eIF2Bα-𝜀), encoded by five genes (*EIF2B1-5*). Mutations reduce the activity of eIF2B (van Kollenburg et al., 2006; Horzinski et al., 2009; Liu et al., 2011).

The eIF2B complex functions as a guanine nucleotide exchange factor (GEF), mediating the exchange of GDP for GTP on eIF2. eIF2-GTP binds to methionine-charged initiator tRNA (Met-tRNA^Met^_i_), thereby forming the ternary complex eIF2.GTP.Met-tRNA^Met^_i_. The ternary complex together with the small ribosomal subunit binds the 5′-end of the mRNA and scans the 5′-untranslated region (5′-UTR) until it encounters a start codon in a suitable context, whereupon translation of the open reading frame (ORF) starts. Simultaneously, GTP on eIF2 is hydrolyzed to GDP which makes the complex inactive (Konieczny and Safer, 1983; Kleijn et al., 1998; Proud, 2001). GEF activity is needed to recharge eIF2 with GTP.

Translation initiation is a complex process, involving multiple eukaryotic initiation factors (eIFs) (Voorma et al., 1994; Sonenberg and Hinnebusch, 2009). Translation initiation efficiency is profoundly influenced by the nucleotides flanking the start codon (usually AUG), the Kozak consensus sequence (Kozak, 1987). Purines at the -3 and +4 position relative to the AUG start codon are most important in determining translation initiation efficiency (Kozak, 1987; Noderer et al., 2014). The architecture of the 5′-UTR with regard to various sequences, structural motifs and length also determines the translation efficiency of an mRNA (Hinnebusch et al., 2016). eIF2B mutations are expected to reduce ternary complex levels and thus overall mRNA translation. However, this reduced activity can actually enhance translation of certain mRNAs with 5′-upstream open reading frames (uORFs) in their 5′-UTR. uORFs can inhibit translation of the main ORF (mORF) and translation of these mORFs depends on translation reinitiation, which is regulated by ternary complex levels (Meijer and Thomas, 2002; Hinnebusch et al., 2016).

A VWM mouse model, homozygous for the Arg191His mutation in the eIF2B𝜀 subunit has been developed, representative of the human disease (*2b5^ho^* mice) (Dooves et al., 2016). The Arg191His mutation corresponds to the Arg195His mutation in patients. This mutation reduces eIF2B activity *in vitro* and gives a severe VWM phenotype (Fogli et al., 2004; Li et al., 2004). Astrocytes in brains of *2b5^ho^* mice are positive for the immaturity marker nestin. *In vitro* experiments show that *2b5^ho^* astrocytes inhibit maturation of wild type (wt) oligodendrocytes (Dooves et al., 2016).

The mechanism by which mutations in eIF2B lead to astrocytic dysfunction and disease remains unclear. Here we aim to improve understanding of the molecular mechanism underlying VWM. We expected that mutant eIF2B would *not* have a general effect on mRNA translation but rather affects translation of a small number of specific mRNAs, leading to disruption of cellular balances and dysfunction of astrocytes in particular. To identify these translational differences, we subjected adult mouse astrocytes to high-resolution quantitative proteomics after a pulse labeling with AHA (L-azidohomoalanine) combined with SILAC (stable isotope-labeling by amino acids in cell culture) (Dieterich et al., 2006; Eichelbaum et al., 2012; Kenney et al., 2016).

## Materials and Methods

### Mice

All experiments were carried out under the Dutch and European law with approval of the local Institutional Animal Care and Use Committee (IACUC) of the VU University (Amsterdam, Netherlands). Wt and *2b5^ho^* animals were used. All animals were weaned at P21 and had *ad libitum* access to food and water. The mice were housed with a 12 h light and dark cycle.

### Astrocyte Culture

Four-month-old mice were sacrificed by cervical dislocation. Brains were taken out and the olfactory bulbs, cerebella and forebrains were removed. Astrocytes from the remaining structures (gray and white matter structures, including striatum, hippocampi and basal nuclei) were isolated. Brain tissue was minced with a scalpel in Hank’s balanced salt solution (HBSS) without magnesium and calcium (Gibco) at 4°C. The tissue was dissociated with a papain solution containing 20 mM PIPES (pH 7.4), 120 mM NaCl, 5 mM KCl, 1.1 mM EDTA, 5.5 mM L-cysteine-HCl, 40 U/ml DNase and 20 U/ml papain for 30 min at 37°C. Cells were plated in poly-L-ornithine (PLO)-coated flasks and cultured in DMEM/F12 with 15% fetal bovine serum (FBS) (Hyclone), 1% sodium pyruvate (Gibco), 100U penicillin, 100 μg/ml streptomycin (Gibco) and 10 μg/ml gentamicin (Gibco). The cells were passaged twice before they were used for experiments. All chemicals were purchased from Sigma–Aldrich unless otherwise stated. Every experiment was replicated in independent cultures derived from different mice (number of experiments is indicated in figure legends as, e.g., *n* = 3).

### Rate of Protein Synthesis Assay

Astrocytes were plated in 6 cm dishes (∼250,000 cells/dish) and cultured until 80–90% confluent. Cells were starved for methionine for 15 min in AHA medium [DMEM/F12 without methionine, lysine, arginine and phenol red (Thermo Fisher Scientific)] supplemented with 91.3 mg/L L-lysine, 147.5 mg/L L-arginine, dialysed FBS and phenol red, after which AHA (Bachem) was added to AHA medium for the indicated time in a final concentration of 2 mM. Cells were harvested in lysis buffer composed of 50 mM Tris pH 7.5, 100 mM NaCl, 0.5 mM EDTA and 0.5% sodium dodecyl sulfate (SDS).

The newly synthesized proteins were labeled using the Click-iT^®^ Protein buffer kit according to the manufacturer’s protocol (Invitrogen). In short, the AHA molecules in the cell lysate were coupled to biotin. Proteins were precipitated by subsequent addition of six volumes of methanol, 1.5 volume of chloroform and 4 volumes of water. The protein pellet was dissolved in 50 mM Tris-HCl pH 7.5 with 1% SDS before subjection to Western blot analysis. Samples were loaded on 12% Criterion^TM^ TGX Stain-Free^TM^ Protein Gels (Bio-Rad), allowing detection of total protein load. Biotinylated AHA-labeled proteins were stained with 400 ng/ml streptavidin (680 nm) and visualized at 700 nm in an Odyssey system (Odyssey^®^ Fc, LI-COR). The amount of AHA-staining was corrected for the total protein load determined by Gel Doc^TM^ EZ System (Bio-Rad).

### Cell Viability Assay

Astrocytes were plated in ½ area 96 well plates (∼5000 cells/well) and cultured for 2 days in AHA-SILAC medium (Thermo Fisher Scientific, custom-made DMEM/F12 without methionine, arginine, lysine and phenol red), with addition of 15% dialyzed serum, 0.005% phenol red, SILAC amino acids (94.2 mg/L [^13^C_6_] L-lysine 152 mg/L [^13^C_6_] L-arginine or 96 mg/L [^13^C_6_, ^15^N_4_] L-lysine and 154.3 mg/L [^13^C_6,_^15^N_4_] L-arginine) and 17.2 mg/L L-methionine. The cells were starved for methionine from 15 min and cultured with 2 mM AHA (Bachem) or methionine overnight in half well area plates. Cell viability (ATP levels, CellTiter-Glo) was measured according to manufacturer’s instructions (Promega). In short, cells were kept at room temperature for 30 min. CellTiter-Glo was added in the same volume as the culture medium. The plate was shaken for 2 min and left standing for 10 min before measuring luminescence with a Victor2 plate reader (PerkinElmer Life Sciences).

### AHA Enrichment and On-Bead Digestion

Astrocytes were plated in 10 cm dishes (∼750,000 cells/dish) and cultured until 80% confluent. The culture medium was replaced with AHA-SILAC medium. Cells were grown for 4 days. SILAC labels were reversed in biological duplicates. Cells were subsequently starved for methionine for 15 min and AHA was added as described in the previous section. Astrocytes were further cultured for 2 h or otherwise indicated. The cells were subsequently washed with cold PBS and lysed in urea lysis buffer (supplied with the Click-IT Protein enrichment Kit, Invitrogen).

For the protein analysis of secreted proteins conditioned medium samples from wt and *2b5^ho^* astrocytes (2 h) were concentrated using Amicon^®^ Ultra-15 centrifuge filter tubes (3 kDa, Merck). Samples were diluted with urea lysis buffer to a volume of 400 μl per sample.

**FIGURE 1:**
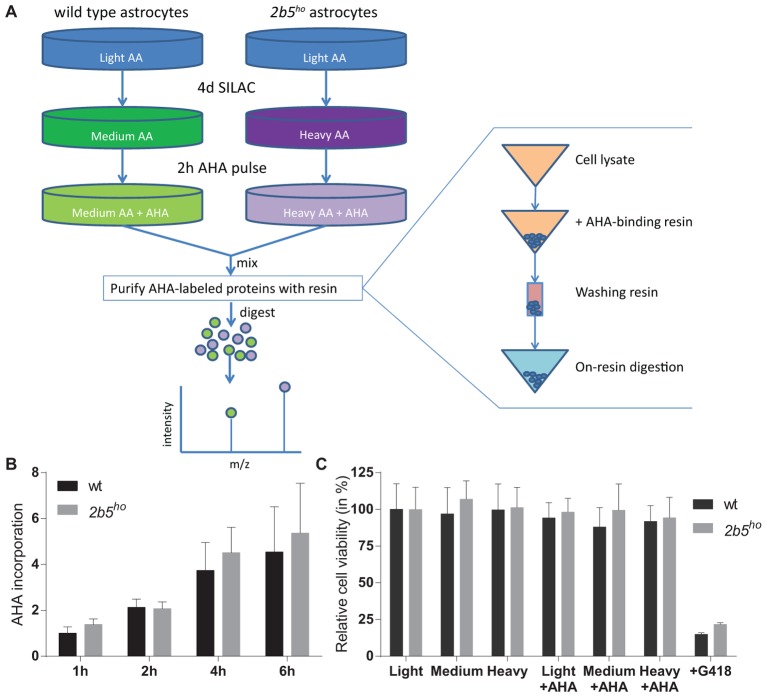
L-azidohomoalanine (AHA) incorporation rates are similar between wt and *2b5^ho^* astrocytes and assay conditions do not affect wt and *2b5^ho^* cell viability. **(A)** Overview of the enrichment protocol. Heavy refers to labeling with [^13^C_6_, ^15^N_4_] L-lysine and [^13^C_6,_^15^N_4_] L-arginine, while medium refers to labeling with [^13^C_6_] L-lysine and [^13^C_6_] L-arginine. **(B)** Astrocytes were treated with AHA for 1–6 h to determine a suitable AHA labeling time to measure newly synthesized proteins as well as confirm a similar AHA incorporation between wt and *2b5^ho^* astrocytes. The graph shows the mean + SD (*n* = 3) and the 1-h-incorporation in wt cells was set to 1. **(C)** Astrocytes were grown in normal (light) or SILAC culture medium (medium or heavy) for 2 days with or without a 16-h AHA pulse. Cell viability was determined by measuring intracellular ATP levels (CellTiter-glo, Promega). As a positive control for the assay, cells were grown in the presence of G418. The graph shows the mean ± SD (*n* = 3).

The AHA-labeled proteins from cell lysates as well as from the conditioned medium were enriched using the Click-iT^®^ Protein Enrichment Kit according to the manufacturer’s protocol (Invitrogen) with some minor modifications. In short, the AHA-labeled proteins were bound to the resin (16 h), following an iodoacetamide treatment and several washing steps. The AHA-labeled proteins bound to the resin were dissolved in 50 mM ammonium bicarbonate with 3 M urea. Digestion was performed at 37°C by adding 0.1 μg Lys-C for 4 h followed by addition of 1 μg trypsin overnight. The peptides were separated from the resin by briefly centrifuging them through a 0.8 ml spin columns (Thermo Fisher Scientific). The flow through contains the peptides. Peptide samples were stored at -80°C until further use. See also **Figure 1** for an overview of the enrichment procedure.

### LC-MS/MS Analysis

Peptides were analyzed using a Q Exactive^TM^ Hybrid Quadrupole-Orbitrap^TM^ Mass Spectrometer (Thermo Fisher Scientific), which was connected to a 1290 Infinity II LC System (Agilent). The trap column was made of C18 (Dr. Maisch Reprosil) material and the analytical column was a 50 cm, 50 μm inner diameter Poroshell C18 (Agilent) column. Both the trap and analytical columns were packed in-house. Solvent A consisted of 0.1% formic acid (Merck) in deionized water (Merck) and Solvent B of 0.1% formic acid in 80% acetonitrile (Biosolve). Peptides were first trapped at 50 μl/min with solvent A and then eluted with solvent B in a 120 min gradient at 100 nl/min: 0–10 min, 100% solvent A; 10.1–105 min, 13–40% solvent B; 105–108 min, 40–100% solvent B; 108–109 min, 100% solvent B; 109–110 min, 0–100% solvent A; 110–120 min, 100% solvent A. The Orbitrap was operated in a data-dependent manner, with the following settings: ESI voltage, 1700 V; inlet capillary temperature 320°C; full-scan automatic gain control (AGC) target, 3 × 10^6^ ions at 35000 resolution; scan range, 350–1500 *m*/*z*; Orbitrap full-scan maximum injection time, 250 ms; MS2 scan AGC target, 5 × 10^4^ ions at 17500 resolution; maximum injection, 120 ms; normalized collision energy, 25; dynamic exclusion time, 30; isolation window 1.5 *m*/*z*; 10 MS2 scans per full scan.

### Data Processing

The LC-MS/MS data were processed with MaxQuant (v1.5.2.8) and MS2 spectra were searched with the Andromeda search engine against the mouse proteome in UniProt (17155 entries, downloaded on 2015-10-27). Enzyme specificity was set to Trypsin/P and two missed cleavages were allowed. SILAC labels (K6/8, R6/10), methionine for AHA substitution and methionine oxidation were set as variable modifications and cysteine carbamidomethylation was set as fixed modification. Minimum peptide length was 7 amino acids. Mass tolerance was set to 20 ppm for peptide masses and 0.6 Da for fragmentation masses. The false discovery rate (FDR) threshold was set to 1% for identifications. Minimal ratio count was set to 2 for protein quantification and the functions “match between runs” and “requantify” were enabled. Data were further analyzed in Perseus (v1.5.0.0). Protein groups were kept for further analysis if they were detected in at least three of the biological replicates. A *t*-test was performed and protein groups were considered significant if the *p*-value was <0.05.

### Signal Peptide Analysis

To assess the presence of an N-terminal signal peptide in the proteins, we subjected the proteins from the proteomic screen to the SignalP 4.1 server (Bendtsen et al., 2004; Petersen et al., 2011). For all UniProt IDs the FASTA files were downloaded from the UniProt website and all Fasta files are automatically subjected to the SignalP 4.1 server. The presence or absence of a signal peptide for each UniProt ID was predicted with the SignalP 4.1 algorithms and the results were saved to Excel. The UniProt IDs linked back to the list of all identified protein names. Biomart was used to identify proteins containing a transmembrane domain. The number of proteins with a signal peptide was calculated for the proteins, the synthesis of which was affected by eIF2B𝜀^Arg191His^ as well as for the other proteins that are not regulated by the same mutation. Next the number of regulated proteins with a signal peptide were subdivided in ‘upregulated’ and ‘downregulated’.

### *In Silico* Analysis of mRNA Features of eIF2B𝜀^Arg191His^-Regulated Proteins

The sequences of the mRNA variants encoding the proteins identified in the AHA-SILAC proteomes were downloaded from the NCBI database including the start and stop codon location. The 5′-UTR features “uORFs, %GC and thermodynamic stability (ΔG in kcal/mol)” were determined for all mRNAs (Babendure et al., 2006). We compared each mRNA characteristic for all proteins found in the AHA-SILAC proteomic screen and compared the characteristics for the proteins regulated by eIF2B𝜀^Arg191His^ and non-regulated proteins. The 5′-UTR length, %GC and ΔG from mRNAs encoding the proteins found in the AHA-SILAC proteomes were analyzed per protein. When a protein is potentially translated from more than 1 mRNA variant (taken from the NCBI database), we counted and analyzed all features of all mRNA variants per protein and plotted all as a proportional representation per protein so that the analysis was not skewed by proteins with multiple variants. 5′-UTR lengths of less than 13 nucleotides were omitted from analyses as AUG codons less than 13 nt from the cap are inefficient to initiate translation (Hinnebusch, 2011). The % of GC in the 5′-UTRs was calculated. To calculate the thermodynamic stability, the free energy (ΔG) of 5′-UTRs was determined using UNAFold^1^, which predicts thermodynamic stability. The uORF was defined as upstream AUG/CUG/GUG/UUG/ACG with a purine (A/G) at the -3 position and a guanine at the +4 position (Ingolia et al., 2011; Ferreira et al., 2013; Noderer et al., 2014). Scripts used for the analyses are found at: https://github.com/LisanneWisse/UORF or https://github.com/LisanneWisse/RefSeq.

### DAVID Analysis

Overrepresentation of specific pathways was analyzed with DAVID [based on gene ontology (GO)-terms] between the proteins that are affected by the eIF2B𝜀^Arg191His^ mutation and all the proteins found in the AHA-SILAC proteome (Huang et al., 2009a,b).

### Cell Lysates for Western Blot Experiments

Astrocytes were plated in 10 cm dishes (∼750,000 cells/dish) and cultured until 80% confluent. Cells were washed with cold PBS, collected by scraping in PBS and pelleted by centrifugation (5 min, 1000 × *g*, 4°C). The method is based on the protocol described^2^. Cells were lysed in harvesting buffer containing 10 mM HEPES pH 7.9, 50 mM NaCl, 0.5 M sucrose, 0.1 mM EDTA, 0.5% Triton, 1 mM DTT, phosphatase inhibitors (0.5 mM activated NaVO_3_, 25 mM β-glycerophosphate, 50 mM NaF) and protease inhibitors (Roche). Lysates were centrifuged (10 min 500 × *g*, 4°C) and the supernatant was used for Western blot analyses.

### Whole Brain Lysates for qPCR and Western Blot Experiments

Mice were sacrificed by cervical dislocation at 4 months of age. Brains were removed, snap-frozen in liquid nitrogen and stored at -80°C until further use. Lysates were prepared by grinding the brain samples with a pestle and mortar under liquid nitrogen. The powder was lysed in cytoplasmic lysis buffer (20 mM Tris pH 7.4, 100 mM KAc, 3 mM MgAc_2_, 2 mM DTT, 1.5% IGEPAL, 1.5% sodium deoxycholate (SODC), 1x HALT (protease and phosphatase inhibitory cocktail, Thermo Fisher Scientific]. The samples were homogenized with a pestle followed by trituration through a 23G needle. The samples were centrifuged (10 min, 10.000 × *g*, 4°C) and the supernatant was aliquoted into pre-chilled microfuge tubes. Protein concentrations were determined using a Quick Start^TM^ Bradford Protein Assay (Bio-Rad). For RNA isolation, TRIzol^TM^ Reagent (Invitrogen) was added to 50 μl of the supernatant and total RNA was isolated as described under RNA isolation and cDNA synthesis.

### Western Blot

For cell lysates approximately 10 μg and for brain lysates 50–60 μg of protein was loaded on a 12% SDS-polyacrylamide gel with 2,2,2-trichloroethanol (TCE) which allow detection of total protein load (Ladner et al., 2004). Proteins were transferred onto a PVDF membrane (Bio-Rad). Membranes were blocked in 5% (w/v) milk powder and stained with the antibody against PROS1 (16910-1-AP, Proteintech) or SLC3A2 (LS-C334231, LSBio) overnight (16 h) at 4°C. Membranes were washed with TBS-Tween20 (0.1%) and incubated with an HRP-conjugated secondary goat anti-rabbit IgG antibody (Dako, P0448) for 2 h at room temperature. The membranes were washed three times with TBS-Tween (0.1%) and once with TBS, incubated with SuperSignal^TM^ West Femto (Thermo Fisher Scientific) and imaged (Odyssey^®^ Fc, LI-COR). Protein expression was corrected for total amount of protein determined by Gel Doc^TM^ EZ System (Bio-Rad).

### RNA Isolation, cDNA Synthesis and qPCR

Astrocytes were washed twice with PBS and collected in TRIzol^TM^ Reagent (Invitrogen). RNA was isolated according to the manufacturer’s protocol. In short, 1/5 volume chloroform was added to the TRIzol^TM^ Reagent. The samples were centrifuged (10 min, 12000 × *g*, 4°C) and the water layer was transferred to a fresh tube. Half a volume of isopropanol and 1/250 volume of linear acrylamide (Ambion) was added and the samples were centrifuged for 20 min at 4°C and 12000 × *g*. The pellet was washed twice with 70% ethanol and resuspended in non-DEPC treated water (Ambion). RNA was precipitated with 175 mM sodium acetate (pH 5.2) and 70% ethanol and incubated for 30 min at -20°C. The samples were centrifuged (30 min, 4°C, 12000 × *g*) and the pellets were washed twice with 70% ethanol and resuspended in non-DEPC-treated water.

RNA quality and quantity were determined by measuring the A260 and A280 (NanoDrop 2000, Thermo Fisher Scientific). cDNA was synthesized in a 20 μl reverse transcription reaction: 1x first strand buffer (Invitrogen), random hexamers (0.02 μg/μl; Qiagen), oligoDT (0.02 μg/μl; Qiagen), dNTPs (1 mM each; Roche), DTT (1 mM; Invitrogen), RNaseOUT (0.25 U/μl; Invitrogen), Superscript III (5 U/μl; Invitrogen) and 1.5 μg total RNA were incubated for 2 h at 50°C. RNAseH (62.5 U/μl; Invitrogen) was added and incubated for 30 min at 37°C followed by 15 min at 70°C.

mRNA levels were determined with qPCR using a LightCycler^®^ 480 II Instrument (Roche). For each 10 μl sample a mixture of LightCycler^®^ 480 SYBR Green I Master (Roche), primers (1 pmol/μl) and cDNA (0.1 μl) was used. Used primers are listed in **Table 1**. *Gapdh* mRNA was used as reference.

**Table 1:** Primers used for qPCR.

Gene name	Forward (5′->3′)	Reverse (5′->3′)
*Gapdh*	GTGCTGAGTATGTCGTGGAG	TCGTGGTTCACACCCATCAC
*Akt*	AAGAAGGAGGTCATCGTCGC	GGTCGTGGGTCTGGAATGAG
*Gas6*	CTAAAACTATCCCCAGACAT	GGTACAAGGACTTCACGCTCT
*Nrxn1*	CCCCACAAAGGAACCCATCA	GTTGGCTAACCCACCTGAGC
*Sorcs1*	GGGACATCAGCCGAGTCATC	AACACCGCCACCAGGATATG
*Fndc3c1*	AGAGCGAGGCTTTTGGAGAA	TGGCACTGTTGGAGGTATTTCA
*Pros1*	TCCCTGGAGGCTACTCTTGTT	AGGTCCAAGGAAAGGCACAC
*Scrn1*	TGTCTGTCTTGCCTCAGAACA	TTTTGGCTGGGTCATCGTCA
*Fgfr1*	GCCAGACAACTTGCCGTATG	TCCGATAGAGTTACCCGCCA
*Txnip*	GTCTCAGCAGTGCAAACAGAC	CCTTCACCCAGTAGTCTACGC
*Ddr1*	CTGCTGCTTCTCATCATCGC	GTCAGCTCCTCCTCCAACAC
*Slc3a2*	GAAAGCTGATGAATGCACCC	CAATTTGCTGCAGGTCAGAG
*Sec31A*	GAGAGTCCTGCTGCTGAAGAG	AGACCGTCAATATCCCCACT

### Constructs

#### Construct to Determine Secretion

The pNL1.3 plasmid (Promega) expresses the nanoluciferase protein with an *N*-terminal signal peptide from interleukin-6 (IL-6; Secluc). The signal peptide promotes secretion of the nanoluciferase into the culture medium. The pNL1.3-*Gapdh* construct contains the promoter including 5′-UTR of *Gapdh*, which was inserted in the pNL1.3 vector using an infusion reaction, according to the manufacturer’s protocol (In-Fusion^®^ HD Cloning Kit, Clontech). The PCR-amplified sequence of the constructs was confirmed by sequence analysis.

#### Constructs to Determine Translation

The pNL1.1 plasmids express the nanoluciferase protein (Nluc). Promoter and 5′-UTR sequences of candidate genes were taken from NCBI and Ensembl databases (**Table 2**). Infusion primers were designed to amplify promoter (approximately 2000 bps upstream of the transcription start site) and 5′-UTR-encoding sequences using the primer design tool on the Clontech website. Internal primers were designed to merge the *Gapdh* promoter region and the 5′-UTR of the candidate.

**Table 2:** Primer sequences used for cloning.

Gene number	Construct name	Primer	Sequence
NM_008084	pNL1.3-*Gapdh*	Infusion FW primer	TGGCCTAACTGGCCGGTACCTGCTGTGTCACTACCGAAGAACAACGAGGAGAAGAT
		Infusion RV primer	GGCTAGCGAGCTCAGGTACCTTTGTCTACGGGACGAGGCTGGCACTGCACAAGAAG
NM_008084	pNL1.1-*Gapdh*	Infusion FW primer	TGGCCTAACTGGCCGGTACCTGCTGTGTCACTACCGAAGAACAACGAGGAGAAGAT
		Infusion RV primer	AGTGTGAAGACCATGGTTTGTCTACGGGACGAGGCTGGCACTGCACAAGAAG
NM_011173	pNL1.1-*Pros1*	Infusion FW primer	TGGCCTAACTGGCCGGTACCAACTGGCTTCTTTGTGGTG
		Infusion RV primer	AGTGTGAAGACCATGGCTGAGAGGATGGCCGGG
NM_019521	pNL1.1-*Gas6*	Infusion FW primer	TGGCCTAACTGGCCGGTACCGGACAGGCACTCTTTGGA
		Infusion RV primer	AGTGTGAAGACCATGGCGAGGCCGGTGCCGGG
NM_027268	pNL1.1-*Scrn1*	Infusion FW primer	TGGCCTAACTGGCCGGTACCCTTTTTTGATTCTGGAAA
		Infusion RV primer	AGTGTGAAGACCATGCTGCCAAGCAGCCGGCT
NM_010206.3	pNL1.1-*Fgfr1*	Infusion FW primer	TGGCCTAACTGGCCGGTACCCAGGGCAAGGATATTGCTA
		Infusion RV primer	AGTGTGAAGACCATGCCAGTTCTGCGGTTAGAG
		Internal primer FW	ACCGCAGCGCCAAGTGAG
		Internal primer RV	CTCACTTGGCGCTGCGGT
NM_011173	pNL1.1-*Pros1* 5′UTR	Infusion FW primer	TGGCCTAACTGGCCGGTACCTGCTGTGTCACTACCGAAGAACAACGAGGAGAAGAT
		Infusion RV primer	AGTGTGAAGACCATGGCTGAGAGGATGGCCGG
		Internal primer FW	GGGTCCAAAGAGAGGGAGGAGCTCGGGCTGGGCCGCGGCAG
		Internal primer RV	TCCTCCCTCTCTTTGGACCCGCCTCATTTT
NM_019521	pNL1.1-*Gas6* 5′UTR	Infusion FW primer	TGGCCTAACTGGCCGGTACCTGCTGTGTCACTACCGAAGAACAACGAGGAGAAGAT
		Infusion RV primer	AGTGTGAAGACCATGGCGAGGCCGGTGCCGGGG
		Internal primer FW	GGGTCCAAAGAGAGGGAGGAACCCGCTGCCTCCTTCACGGC
		Internal primer RV	TCCTCCCTCTCTTTGGACCCGCCTCATTTT
NM_010206.3	pNL1.1-*Fgfr1* 5′UTR	Infusion FW primer	TGGCCTAACTGGCCGGTACCTGCTGTGTCACTACCGAAGAACAACGAGGAGAAGAT
		Infusion RV primer	AGTGTGAAGACCATGCCAGTTCTGCGGTTAGA
		Internal primer FW	GGGTCCAAAGAGAGGGAGGAGCACAGCGCTCGGAGCGCTCC
		Internal primer RV	GGAGCGCTCCGAGCGCTGTGCTCCTCCCTCTCTTTGGACCC
NM_020252.3	pNL1.1-*Nrxn1* 5′UTR	Infusion FW primer	TGGCCTAACTGGCCGGTACCTGCTGTGTCACTACCGAAGAACAACGAGGAGAAGAT
		Infusion RV primer	AGTGTGAAGACCATGCTCGGGGCTGGGGTGCG
		Internal primer FW	GGGTCCAAAGAGAGGGAGGACCTTTTTCCCTCTCCTCCTCC
		Internal primer RV	GGAGGAGGAGAGGGAAAAAGGTCCTCCCTCTCTTTGGACCC
NM_021377	pNL1.1-*Sorcs1* 5′UTR	Infusion FW primer	TGGCCTAACTGGCCGGTACCTGCTGTGTCACTACCGAAGAACAACGAGGAGAAGAT
		Infusion RV primer	AGTGTGAAGACCATGTCTGGAGCGTAGAGAAG
		Internal primer FW	GGGTCCAAAGAGAGGGAGGAAGCCTGGGCGAGCGGCAGGCA
		Internal primer RV	TCCTCCCTCTCTTTGGACCCGCCTCATTTT
NM_001198833	pNL1.1-*Ddr1* 5′UTR	Infusion FW primer	TGGCCTAACTGGCCGGTACCTGCTGTGTCACTACCGAAGAACAACGAGGAGAAGAT
		Infusion RV primer	AGTGTGAAGACCATGGCTCTCCGGGGCGGACC
		Internal primer FW	GGGTCCAAAGAGAGGGAGGATGGCTCCTCTCCCCGGAACAG
		Internal primer RV	TCCTCCCTCTCTTTGGACCCGCCTCATTTT
NM_011173	pNL1.1-*Pros1* promoter	Infusion FW primer	TGGCCTAACTGGCCGGTACCAACTGGCTTCTTTGTGGTG
		Infusion RV primer	AGTGTGAAGACCATGGTTTGTCTACGGGACGAGGCTGGCACTGCACAAGAAG
		Internal primer FW	TGGCTGCTCCGCCCGCCCGCGGGGAAATGAGAGAGGCCCA
		Internal primer RV	GCGGGCGGGCGGAGCAGCCA

The promoter and 5′-UTR sequences were amplified using the infusion primers and the overlapping internal primers on gDNA from mouse liver or cDNA from cultured mouse astrocytes (**Table 2**) according to the manufacturer’s protocol (ClonAmp, In-Fusion HD, Clontech). For the chimeric constructs comprising the murine *Gapdh* core promoter and 5′-UTR of the candidate mRNA a triple infusion reaction was performed with the pNL1.1 vector, the amplified promoter product and the amplified 5′-UTR product (**Table 2**). All promoter and 5′-UTR sequences were inserted into the pNL1.1 vector (Promega) using an infusion reaction according to the manufacturer’s protocol (In-Fusion^®^ HD Cloning Kit, Clontech). The resulting plasmids are listed in **Table 2**. PCR-amplified sequences of all constructs were confirmed by sequence analysis.

#### Construct Used As Internal Control

The pGL3 plasmid expresses the firefly luciferase protein (Fluc). The murine *Gapdh* promoter and 5′-UTR were digested from the pNL1.1 vector with KpnI and NcoI, purified from agarose gel (High Pure PCR Cleanup, Roche) and inserted into an empty pGL3 vector digested with the same restriction enzyme using a T4 DNA ligase (Promega). The resulting plasmid pGL3-*Gapdh* was used as internal standard in transfection studies. PCR-amplified sequence of the constructs was confirmed by sequence analysis.

### Measurement of Secretion and Secretory Pathway Flux

Astrocytes were plated in half-area 96 well plates (∼3000 cells/dish, CELLSTRAR) to 80% confluency. pNL1.3-Gapdh (80 ng) was transfected into the cells using 0.24 μl FuGENE^®^ 6 according to the manufacturer’s protocol (Promega). Luciferase activity in cells and culture medium was measured using the protocol of Promega and a Wallac 1420 Victor2 Microplate Reader (Perkin Elmer). The ratio of extracellular/intracellular luciferase activity determines the relative secretion.

### Transfections

Astrocytes were plated in half-area 96 well plates (∼3000 cells/dish, CELLSTAR). Each pNL1.1 promoter/5′-UTR construct (40 ng) was co-transfected with internal control pGL3-*Gapdh* (20 ng) using FuGENE^®^ 6 Transfection Reagent (0.24 μl) according to the manufacturer’s protocol. Luciferase activity was measured using the protocol of Promega and a GloMax^®^ Discover System (Promega). The ratio Nluc:Fluc determines the expression of Nluc corrected for well-by-well differences.

### Metabolomics

Astrocytes were cultured in 6 cm dishes (∼250,000 cells/dish) until 70% confluent. Medium was replaced 72 and 24 h before harvesting. At the time of harvesting culture medium was removed from the cells and stored at -80°C until analysis. Cells were washed with ice-cold PBS and metabolites were extracted from cells in 0.5 ml lysis buffer containing methanol/acetonitrile/dH_2_O (2:2:1). Samples were spun at 16.000 × *g* for 15 min at 4°C. Supernatants were collected for LC-MS analysis. 10 μl of conditioned medium was added to 1 mL of lysis buffer containing methanol/acetonitrile/H_2_O (2:2:1) and prepared as above.

LC-MS analysis was performed on an Exactive mass spectrometer (Thermo Fisher Scientific) coupled to a Dionex Ultimate 3000 autosampler and pump (Thermo Fisher Scientific). The MS operated in polarity-switching mode with spray voltages of 4.5 and -3.5 kV. Metabolites were separated using a SeQuant^®^ ZIC^®^-pHILIC HPLC Columns (2.1 mm × 150 mm, 5 μm, guard column 2.1 mm × 20 mm, 5 μm; Merck) using a linear gradient of acetonitrile and eluent A [20 mM (NH_4_)_2_CO_3_, 0.1% NH_4_OH in ULC/MS grade water (Biosolve)]. Flow rate was set at 150 μl/min. Metabolites were identified and quantified using LCQUAN^TM^ Quantitative Software (Thermo Fisher Scientific) on the basis of exact mass within 5 ppm and further validated by concordance with retention times of standards. Metabolites were quantified using LCQUAN^TM^ Quantitative Software (Thermo Fisher Scientific). Peak intensities were normalized based on median peak intensity.

### Statistical Analyses

The program Factor was used to correct for differences between experiments (qPCR, Western blot, cell viability) but not between other conditions (genotype, treatments) (Ruijter et al., 2006). Statistical analysis of qPCR, Western blot and cell viability experiments were performed with a two way ANOVA followed by a Sidaks multiple comparison test, using GraphPad Prism software. Statistical analysis of the transfection data was performed using a *T*-test per construct.

A Chi-square analysis was used to measure significant differences in the presence of signal peptides, transmembrane regions and SP-targeting proteins as well as for the analysis on the 5′-UTR features using GraphPad Prism software.

The secretion assay was statistically tested with a multilevel analysis in SPSS. Differences were significant when *p* < 0.05.

## Results

### AHA-SILAC Incorporation Is Not Affected by Mutations in eIF2B and Does Not Affect Astrocyte Viability

L-azidohomoalanine incorporation was measured in wt and *2b5^ho^* astrocytes to investigate if the general protein synthesis rate is affected by the homozygous Arg191His mutation in eIF2B𝜀 (**Figure 1**). Label incorporation increased linearly for a period of 4 h (**Figure 1B**). The incorporation occurred with similar kinetics in *2b5^ho^* and wt astrocytes (**Figure 1B**), indicating that the eIF2B𝜀 Arg191His mutation did not significantly affect protein synthesis rate. The viability of both wt and *2b5^ho^* astrocytes was not influenced by AHA or SILAC treatment (**Figure 1C**). Expression of stress-related mRNAs was unaffected by the AHA-labeling protocol (Supplementary Figure 1). These results allow further proteomic analyses with an AHA incorporation pulse of 2 h.

### Pulsed Labeling Proteomics of Astrocytes Reveals 80 Proteins Regulated by the eIF2B𝜀 Arg191His Mutation

**FIGURE 2:**
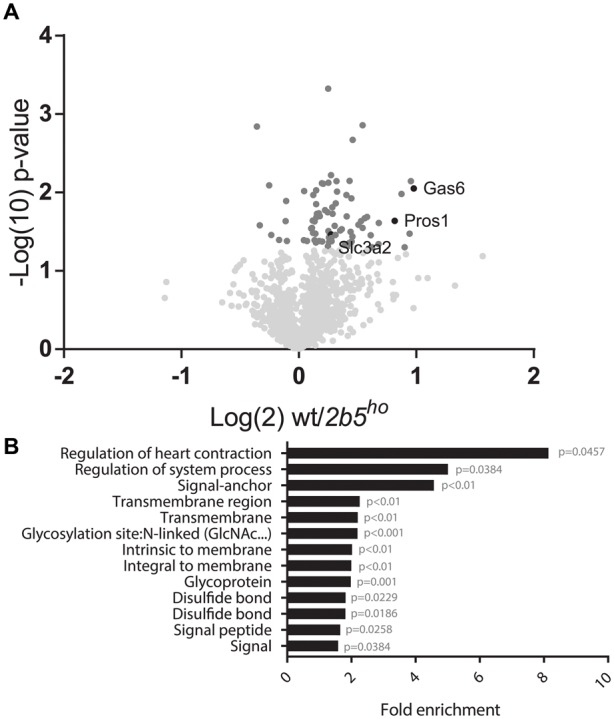
Volcano plot of all 1240 proteins from the proteomic screen and DAVID analysis of the 80 proteins regulated by eIF2B𝜀^Arg191His^ identifies a difference in proteins that migrate through the secretory pathway. **(A)** In the volcano plot the proteins in darker gray have a *p*-value < 0.05 and are considered significantly differently synthesized between wt and *2b5^ho^* astrocyte cultures. The *p*-values are plotted as Log(10) and *p* < 0.05 corresponds to -Log10 > 1.12. The fold change between wt and *2b5^ho^* is plotted as Log(2) and a fold change of >2 corresponds to Log(2) > 1. 80 proteins are significantly different between wt and *2b5^ho^* astrocytes of which 72 are increased and 8 are decreased in *2b5^ho^* astrocytes. The proteins SLC3A2, PROS1, and GAS6 are highlighted in black. **(B)** The figure shows the overrepresentation analysis (*p*-value < 0.05) between the significantly different proteins with the proteins found in the proteome as a background. The pathway analysis reveals an overrepresentation of proteins with a signal peptide, transmembrane domain, *N*-linked glycosylation site(s) and/or disulfide bond(s). These terms together pinpoint targeting of proteins to the secretory pathway, some of which are post-translationally modified, as being affected by this mutation.

The AHA proteomic labeling approach was performed to identify and quantify proteins that are differentially translated in *2b5^ho^* astrocytes. SILAC-labeled wt and *2b5^ho^* astrocytes were subjected to AHA labeling for 2 h. After AHA labeling cells were harvested and subjected to bead-based enrichment using a Click-iT chemistry approach. Bound proteins were digested and resulting peptides were analyzed by LC-MS/MS. In four biological replicates, we identified a total of 2888 proteins across both wt and *2b5^ho^* astrocytes. 1240 proteins were detected in at least 3 out of 4 biological replicates for both wt and *2b5^ho^* astrocytes and used for further analysis. Accumulation of 72 proteins was increased and of eight proteins decreased in the *2b5^ho^* AHA-proteome (*p* < 0.05, student’s *T*-test) (**Figure 2A** and Supplementary File 1).

### eIF2B𝜀^Arg191His^-Regulated Proteins Localize Predominantly to the Plasma Membrane or Extracellular Space

**FIGURE 3:**
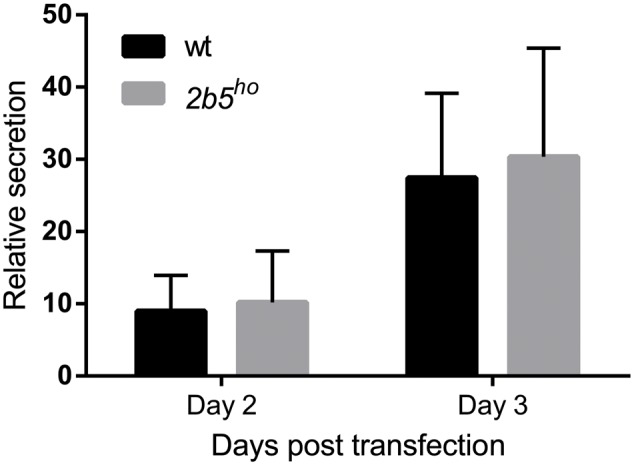
The secretion of Secluc nanoluciferase via the secretory pathway is not affected in *2b5^ho^* astrocyte cultures. Astrocytes were transfected with a luciferase reporter construct that expresses nanoluciferase fused to the IL-6 signal peptide. Luciferase activity was measured 2 and 3 days post transfection in cell lysates and cell culture medium. The ratio of extracellular:intracellular luciferase activity was determined as a measure for secretion through the secretory pathway. In the absence of the signal peptide, the extracellular luciferase activity was similar to the background signal (Supplementary Figure 2). The graph shows the mean ± SD (three independent cultures, six wells per culture).

We performed a DAVID pathway analysis to investigate if the eIF2B𝜀^Arg191His^-regulated proteins share common functions, localizations or other features. This analysis showed enrichment for *N*-terminally glycosylated proteins, membrane proteins, proteins with disulfide bonds as well as proteins that contain a signal peptide among the set of eIF2B𝜀^Arg191His^-regulated proteins (**Figure 2B**). This outcome suggests that many regulated proteins follow the secretory pathway. Since signal peptides and transmembrane domains are associated with targeting to the secretory pathway (von Heijne, 1990; Petersen et al., 2011; Borgese, 2016), we tested all 1240 identified proteins for signal peptides or transmembrane domains. Signal peptides were found in 237 proteins and transmembrane domains in 177. The number of proteins predicted to pass through the secretory pathway was significantly enriched in the *2b5^ho^* proteome (**Table 3**). 27 out of the 80 regulated proteins harbor a signal peptide and 24 have at least one transmembrane domain; 13 of the proteins harbor both a signal peptide and a transmembrane domain (in total 38 proteins out of 80). Intriguingly, the proteins annotated as migrating through the secretory pathway were all upregulated in *2b5^ho^* astrocytes. It is possible that these proteins accumulate more in *2b5^ho^* astrocytes during the 2-h labeling pulse due to a secretory pathway flux difference and not to an actual increase in translation rate. To investigate this, we analyzed the AHA-enriched secretome from wt and *2b5^ho^* cultures after a 2-h AHA labeling. We detected 22 labeled proteins in the secretome, of which three (APOE, CST3 and POSTN) were significantly reduced in the *2b5^ho^* astrocyte-conditioned medium. Intracellularly, APOE and POSTN were not changed by eIF2B𝜀^Arg191His^. CST3 was significantly increased intracellularly in *2b5^ho^* astrocytes. No other protein from the original 27 eIF2B𝜀^Arg191His^-regulated proteins with a signal peptide was detected in the secretomes of wt and *2b5^ho^* astrocytes. To further address the hypothesis that *2b5^ho^* astrocytes have an altered secretory pathway flux, we evaluated the flux of proteins migrating through the secretory pathway with a reporter assay. The assay showed robust secretion of the Secluc reporter (Supplementary Figure 2) and secretion differences between wt and *2b5^ho^* astrocytes were not detected (**Figure 3**).

**Table 3:** Analysis of the proteins that are synthesized and migrate in the secretory pathway (SP).

AHA-SILAC data set^∗^)	% of proteins with a signal peptide (absolute #)	% of proteins with a transmembrane domain (absolute #)	% of SP-targeting proteins (absolute #)
All proteins (1240)	19.1% (237)	14.3% (177)	25.6% (317)
Regulated (80)	33.8% (27)	30.0% (24)	47.5% (38)
Upregulated (72)	37.5% (27)	33.3% (24)	52.7% (38)
Downregulated (8)	0% (0)	0% (0)	0% (0)

*^∗^All proteins, proteins measured in wt and *2b5^*ho*^* astrocytes; regulated, proteins that differ significantly in intracellular accumulation between wt and *2b5^*ho*^* astrocytes (divided in upregulated and downregulated).*

### Validation of eIF2B𝜀^Arg191His^-Regulated Proteins

**FIGURE 4:**
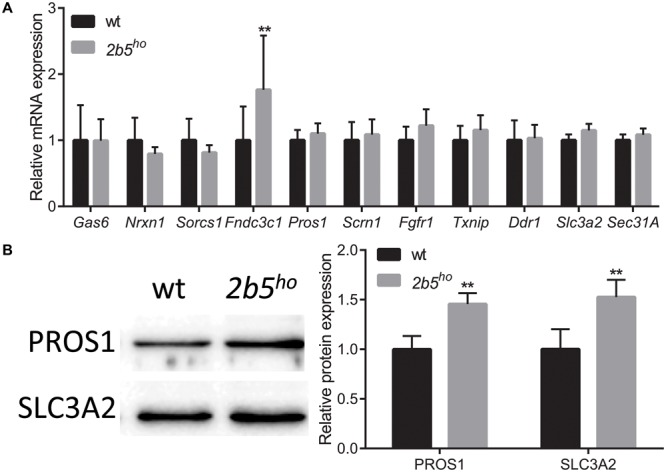
mRNA and protein levels of candidate proteins in astrocyte cultures. **(A)** mRNA levels of the investigated candidate were investigated with qPCR. The mRNA levels were similar between wt and *2b5^ho^* astrocytes (*n* ≥ 5), with the exception of the *Fndc3c1* mRNA, which was increased in *2b5^ho^* astrocytes. **(B)** The total protein levels of PROS1 and SLC3A2 were determined by Western blot. The graph shows the mean ± SD. The protein levels were increased in *2b5^ho^* cultures compared to wt cultures (*n* ≥ 3). ^∗∗^*p* < 0.01. Post-translational modifications were overall not affected in *2b5^ho^* astrocytes. Western blot analyses did not show whether PROS1 in astrocytes was post-translationally modified or not. Staining of all proteins (loading control) is shown in Supplementary Figure 4.

To investigate if the proteins that were found in the screen are indeed differentially expressed at the translational level, we selected eleven candidates that are either increased or decreased in wt vs. *2b5^ho^* cultures. We quantified their mRNA levels to discriminate whether differences in protein amounts are regulated at the transcriptional or the translational level. Only one of the selected candidates differed in mRNA expression between wt and *2b5^ho^* astrocytes (**Figure 4A**), suggesting that the increased accumulation of the other 10 proteins was not due to increased transcription or mRNA stability. Of the 11 candidates two were further investigated at the protein level by Western blot. We found that PROS1 and SLC3A2 were indeed significantly increased at the total protein level (**Figure 4B**). This result confirms the data from the proteomic screen and also demonstrates increased accumulation of these candidate proteins.

We next investigated several targets in mouse brain. At the mRNA level seven out of nine targets were similar between wt and *2b5^ho^* brains (Supplementary Figure 3A). Two proteins were further assessed using Western blot (Supplementary Figure 3B). SLC3A2 protein abundance was also increased in brain lysates; however, this correlated with increased *Slc3a2* mRNA levels, suggesting transcriptional regulation in brain. PROS1 protein abundance was also increased in brain lysates, but *Pros1* mRNA abundance was not. This finding suggests translational upregulation of PROS1 both in cell culture and brain.

### *In Silico* Analysis of mRNAs

**FIGURE 5:**
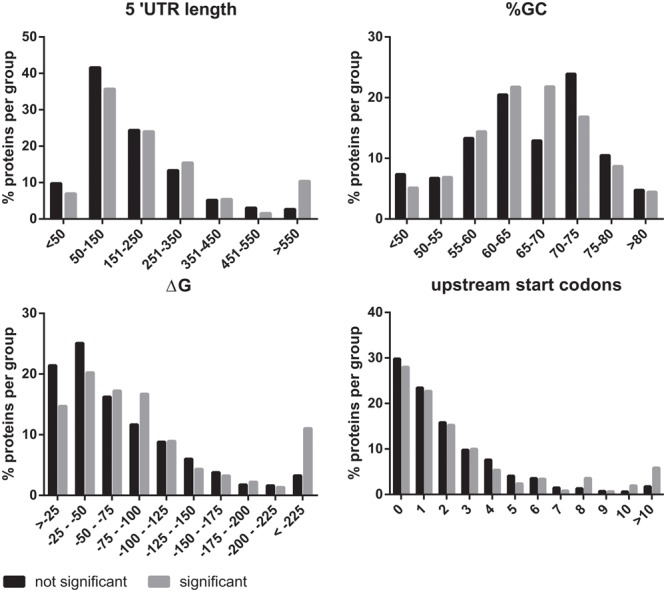
*In silico* 5′UTR analysis of the eIF2B^Arg191His^-regulated proteins. The 5′UTRs of the mRNAs encoding the proteins regulated by eIF2B𝜀^Arg191His^ mRNAs are significantly longer than of those encoding non-regluated proteins. They have an overall similar percentage GC content to the non regulated proteins. However, they seem to tend to higher ΔG’s. These findings combined are indicative of relatively structured 5′-UTRs in the mRNAs of eIF2B𝜀^Arg191His^-regulated proteins. The number of uORFs is similar between the significant and not significant proteins, though mRNAs with very high numbers (8 or more) of predicted uORFs are overrepresented in the group of significantly altered proteins.

eIF2B plays an essential role in the regulation of protein synthesis. For this reason, we investigated if the mRNAs of the eIF2B𝜀^Arg191His^-regulated proteins share specific features that could explain the translational regulation by eIF2B. We investigated the 5′-UTRs for length, structural stability (%GC and ΔG) and number of uORFs with a Kozak sequence. The 5′-UTR length was significantly increased for mRNAs encoding eIF2B𝜀^Arg191His^-regulated proteins (**Figure 5**). Of note, the mRNAs for the eight proteins *decreased* in the eIF2B𝜀^Arg191His^-regulated proteome have relatively short 5′-UTRs (29-343 bases, median 133). This analysis showed that the eIF2B𝜀^Arg191His^ mutation differentially influences expression of proteins translated from mRNAs with *short* 5′-UTRs (<150 bases), which were underrepresented, compared to those with lengthy 5′-UTRs (>550 nucleotides), which were overrepresented; those translated from mRNAs with short 5′-UTRs, if regulated, tend to go down in expression in the mutant cells. The %GC in the 5′-UTRs did not significantly differ between eIF2B𝜀^Arg191His^-regulated proteins and other proteins (**Figure 5**). The thermodynamic stability (ΔG in kcal/mole) of the 5′-UTR seemed to be higher for mRNAs encoding the eIF2B𝜀^Arg191His^-regulated proteins, although not significantly (**Figure 5**). The overall number of uORFs did not significantly differ for mRNA encoding the eIF2B𝜀^Arg191His^-regulated proteins compared to the non-regulated proteins (**Figure 5**). However, mRNAs with high numbers of uORFs (8 or more) were clearly overrepresented in the group of significantly altered proteins.

### Translation Efficiency Was Assessed in Reporter Assays

**FIGURE 6:**
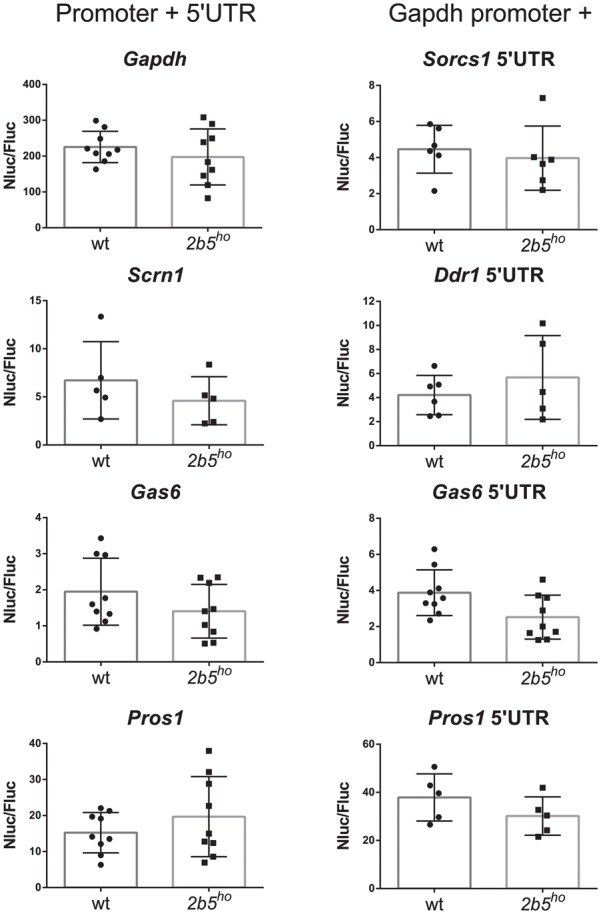
*In vitro* analyses of candidate gene expression regulation do not reveal difference in promoter or 5′-UTR efficiency in wt and *2b5^ho^* astrocytes. pNL1.1- *Scrn1*, -*Gas6* and -*Pros1* constructs and pNL1.1-*Sorcs1*, -*Ddr1*, -*Gas6* and -*Pros1* 5′UTR constructs were transfected into wt and *2b5^ho^* astrocytes. Nanoluciferase activity was measured 3 days post transfection and corrected for firefly luciferase activity expressed from the pGL3-*Gapdh* internal standard. Every culture is shown as a single dot and bars represent the mean ± SD (*n* = 5–9). Replacement of the authentic *Pros1* or *Gas6* promoter by the *Gapdh* promoter increased the nanoluciferase expression by approximately twofold. pNL1.1-*Gapdh* was used as reference non-regulated promoter.

We investigated the potential translational regulation of some eIF2B𝜀^Arg191His^-regulated proteins using reporter constructs encoding nanoluciferase driven by promoter sequences (including the 5′-UTR) of the candidates GAS6, PROS1, SCRN1 and FGFR1. A *Gapdh* promoter construct was included as non-regulated control. Transfection of each construct yielded similar luciferase expression in wt and *2b5^ho^* cultures (**Figure 6**). Transfection of the pNL1.1-*Fgfr1* promoter construct did not yield a reliable level of nanoluciferase (approximately two–threefold over the background signal in non-transfected cells) and was therefore omitted from analysis. The levels of nanoluciferase activity expressed with the other constructs were still low in comparison to the *Gapdh*-promoter-driven expression. Because expression could be increased by replacing the candidate promoter with the *Gapdh* promoter, we therefore constructed *Gapdh*-candidate chimeras. With these chimeras we tested the 5′-UTR efficiency of candidates GAS6, SORCS1, PROS1, FGFR1, NRXN1, and DDR1 in wt and *2b5^ho^* cultures. Neither the pNL1.1-*Fgfr1*-5′-UTR nor the pNL1.1-*Nrxn1*-5′-UTR constructs yielded quantifiable nanoluciferase expression. These constructs were omitted from further analyses. The expression from the chimeric promoter-5′-UTR constructs of *Gas6* and *Pros1* was increased by the *Gapdh* core promoter in both wt and *2b5^ho^* cells. Still, none of these constructs yielded an increased nanoluciferase expression in *2b5^ho^* compared to wt astrocytes (**Figure 6**).

### Metabolic Screen of Astrocytes Conditioned Medium and Lysates

**FIGURE 7:**
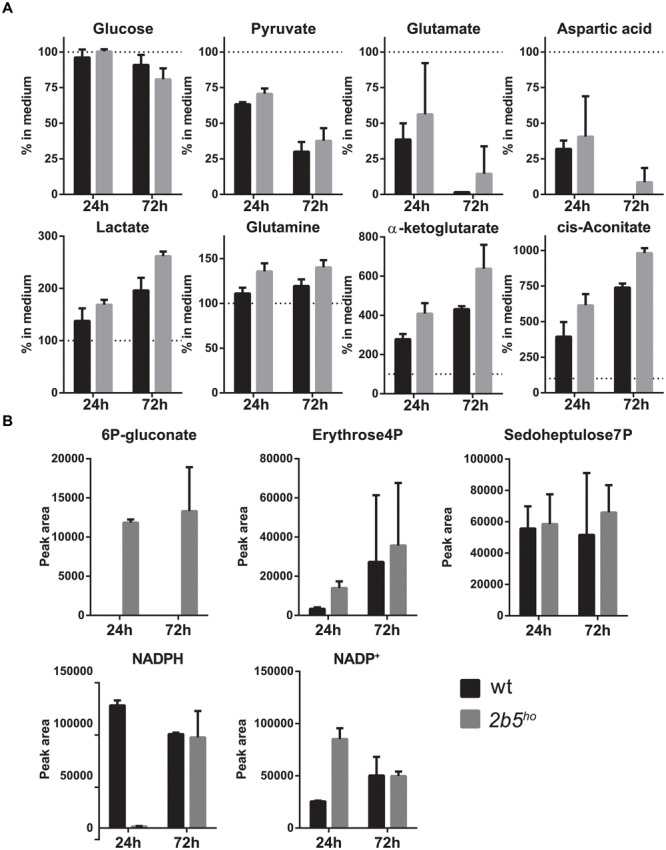
Intracellular and extracellular metabolic analysis after 24 and 72 h of culture showed increased levels of intracellular levels of a pentose phosphate pathway intermediate (6P-gluconate). The astrocytes were cultured until 70% confluent. Media of the cultures were replaced with fresh medium. The cells as well as the media were collected after 24 and 72 h. The graphs show the mean ± SD. **(A)** The metabolite concentrations in the culture media of wt and *2b5^ho^* astrocytes were compared to unconditioned media (*n* = 2). Glucose levels and pyruvate levels in conditioned medium of wt and *2b5^ho^* astrocytes were decreased as well as glutamate and aspartate levels. Lactate, glutamine, *cis*-aconitate and α-ketoglutarate were increased in the conditioned medium of both cultures. **(B)** 6P-gluconate levels were consistently detected in *2b5^ho^* cultures but not in wt cultures (24 and 72 h after replacing the culture medium). Four other metabolites of the pentose phosphate pathway were detected in the metabolic screen (erythrose-4P and sedoheptulose-7P, NADPH and NADP^+^), but these were not consistently altered between wt and *2b5^ho^* cultures.

Because the proteomic data only gave small differences, the VWM phenotype remained difficult to explain. Tiny differences in protein levels and post-translational modifications occasionally lead to metabolic shifts. Thus we checked whether they affected cellular metabolism, using a mass spectrometry-based metabolic screen. We measured intra- and extracellular metabolites 24 and 72 h after replacing the culture medium. We looked at general energy consumption by measuring the uptake and secretion of metabolites from the culture medium (**Figure 7A**). As expected, both cultures had taken up glutamate (Martin-Jimenez et al., 2017) and aspartate (which use the same transporters) (Bender et al., 1997), reflecting specific astrocyte function. The uptake of glutamate and aspartate did not influence their intracellular levels (Supplementary File 2). Also, both wt and *2b5^ho^* cultures showed uptake of pyruvate. Astrocytes convert pyruvate into lactate and glutamate/aspartate into glutamine, *cis*-aconitate and α-ketoglutarate (Martin-Jimenez et al., 2017). Indeed, the wt as well as the *2b5^ho^* cultures secreted lactate, glutamine, *cis*-aconitate and α-ketoglutarate (**Figure 7A**). In addition, we investigated intracellular metabolites (**Figure 7B**). 6P-gluconate, an intermediate of the pentose phosphate pathway (PPP), was detected in *2b5^ho^* but not in wt cells at both time points (**Figure 7B**). A fluctuation over time in NADPH levels was observed. The remaining metabolites tested did not show consistent differences over time between wt and *2b5^ho^* astrocytes (**Figure 7B** and Supplementary File 2).

## Discussion

Here we aimed to identify whether specific proteins are translationally regulated by eIF2B𝜀^Arg191His^ in astrocytes. We used primary astrocyte cultures of the *2b5^ho^* mouse model. Both wt and *2b5^ho^* cells display an astrocyte morphology, synthesize CD44, nestin and vimentin (Supplementary Figure 5 and File 1), supporting astrocyte identity. We first established that cultures of primary astrocytes are suitable for sensitive labeling of newly synthesized proteins. Consistent with previous studies (Howden et al., 2013; Genheden et al., 2015), we found that AHA-labeling did not affect murine astrocyte viability for at least 16 h. No differences in AHA-labeling efficiency were observed between wt and *2b5^ho^* astrocytes. These results confirm that the pulsed AHA-SILAC labeling protocol is suitable to study differences in proteins synthesized in wt and *2b5^ho^* astrocytes, enabling us to investigate whether the Arg191His mutation in eIF2B𝜀 affects translation of specific mRNAs.

We identified 80 proteins that accumulate differentially in wt vs. *2b5^ho^* astrocyte cultures upon a 2 h AHA pulse. The majority (72 out of 80) is upregulated in *2b5^ho^* astrocytes. We investigated the proteomic results by qPCR (11 candidates) and Western blot (4 candidates) (**Figure 4**). The tested antibodies detecting PROS1 and SLC3A2 showed consistent differences between wt and *2b5^ho^* while TXNIP gave variable results and SCRN1 was not detected. Changes observed at the proteomic level were not due to altered mRNA levels, indicating that differences arose at the translational level. PROS1 and SLC3A2 protein levels were also increased in *2b5^ho^* mouse brain lysates, supporting the findings in astrocyte cultures. Of these, *Pros1* was not increased at the mRNA level.

Our analyses indicate significant enrichment for proteins containing a signal peptide and motifs for *N*-glycosylation and disulfide bond formation in *2b5^ho^* astrocytes (**Figure 2B**). These motifs highlight involvement of the endoplasmic reticulum (ER), the first organelle of the secretory pathway (Farhan and Rabouille, 2011). Approximately 50% of the eIF2B𝜀^Arg191His^-regulated proteins were predicted to use the secretory pathway, suggesting an increased flux in *2b5^ho^* astrocytes. The analysis of the secretome after 2 h AHA labeling as well as a standardized secretion assay did not indicate a difference in general secretion between wt and *2b5^ho^* astrocytes. The increased amount of proteins using the secretory pathway observed in the proteomic screen may still indicate a deregulation of ER function. The secreted luciferase reporter used in the standardized secretion assay was not modified in the ER. Protein modifications could be further investigated by expressing a reporter protein that is, e.g., *N*-glycosylated, actively folded by chaperones and/or subjected to extensive disulfide bond formation in ER and Golgi. However, transfections with such reporter constructs probably do not yield protein levels sufficient to quantify the small differences in these processes. Overexpression of such reporters will overburden the ER and induce an unfolded protein response, compromising ER function, preventing reliable conclusions.

Next we investigated if the mRNAs for eIF2B𝜀^Arg191His^-regulated proteins contain uORFs as reduced eIF2B activity increases translation of mRNAs with one or more uORFs in the 5′-UTR (Meijer and Thomas, 2002). *In silico* analyses of the 5′-UTR sequences of the candidates did not reveal obvious overall differences in uORF numbers. Nonetheless, mRNAs with high numbers of uORFs (8 or more) were clearly overrepresented in the group of significantly altered proteins (**Figure 5**). Thus, when eIF2B activity is reduced, protein synthesis of those mRNAs may be enhanced due to inefficient initiation on inhibitory uORFs, stimulating initiation on the “normal” start codon of the mORF (Meijer and Thomas, 2002). Our analyses highlighted some overrepresentation for mRNAs with (especially) long 5′-UTRs and probably higher ΔG in *2b5^ho^* astrocytes (**Figure 5**). These length and ΔG observations suggest greater dependence on RNA helicase activity during translation initiation of eIF2B𝜀^Arg191His^-regulated proteins (Sen et al., 2016). To further characterize the 5′-UTR efficiency in wt and *2b5^ho^* astrocytes, we performed transient transfection assays for several candidates, some of which were longer than 700 nucleotides (*Fgfr1* and *Nrxn1*). Unfortunately, transfection of the *Fgfr1* and *Nrxn1* constructs did not yield reliable expression, which precluded testing whether they are translated more efficiently in *2b5^ho^* than in wt astrocytes. The transfection assay is not robust enough to detect differences that were picked up with the pulsed AHA-SILAC proteomics and *in silico* 5′UTR analyses.

PROS1 was shown to be upregulated in both *2b5^ho^* astrocytes and brain without increased mRNA level. In brain, PROS1 is expressed by astrocytes and microglia (Zhang et al., 2014). Interestingly, PROS1 and GAS6 (which was also found to be upregulated) bind the same class of receptors (Hafizi and Dahlback, 2006). PROS1 and GAS6 enhance myelination and support oligodendrocyte survival in mice *in vitro* and *in vivo* (Tsiperson et al., 2010; Gruber et al., 2014; Goudarzi et al., 2016; Akkermann et al., 2017). Recently, PROS1 was described to function in neural stem cells (NSCs) as a regulator for NSC quiescence, proliferation and NSC development into neurons or astrocytes (Zelentsova et al., 2016).

Other proteins enriched in the eIF2B𝜀^Arg191His^-regulated proteome are implicated in astrocyte development. For example NRXN1 (Zeng et al., 2013) and SORCS1, that transports NRXN1, can induce differentiation toward astrocytes (Savas et al., 2015). Moreover TGFβ2 (Park et al., 2012) and its binding partners, ERBIN and latent TGFβ-binding protein 3 (LTBP3) are increased during astrocyte differentiation (Robertson et al., 2015). All these results suggest that *2b5^ho^* astrocytes have a slightly altered differentiation state as shown before in *2b5^ho^* mice and VWM patients (Bugiani et al., 2011; Dooves et al., 2016).

To investigate other functional changes, we compared the metabolomes of wt and *2b5^ho^* cells. We detected glutamate uptake in both cultures, which is a typical function of astrocytes, (Martin-Jimenez et al., 2017). This observation further confirms the astrocytic identity of the cultured cells. The most consistent difference observed was an increase in 6P-gluconate, an intermediate of the PPP. The PPP shunt is an alternative route for the metabolism of glucose which can supply ribose for nucleotide production in the non-oxidative part and NADPH in the oxidative part of the pathway. The reducing equivalents provided by NADPH are used both for biosynthesis and repair of oxidative damage (Stincone et al., 2015). The oxidative intermediate 6P-gluconate was increased in *2b5^ho^* astrocytes while the non-oxidative intermediates sedoheptulose 7-phosphate and erythrose 4-phosphate were not consistent over time. These results suggest an increased activity of the PPP, especially at earlier time points. Interestingly, the observed PPP signature has been found in lung cancer cells in response to reduced 6P-gluconate dehydrogenase (6PGDH) activity leading to reactive oxygen species (ROS) production (Patra and Hay, 2014).

**FIGURE 8:**
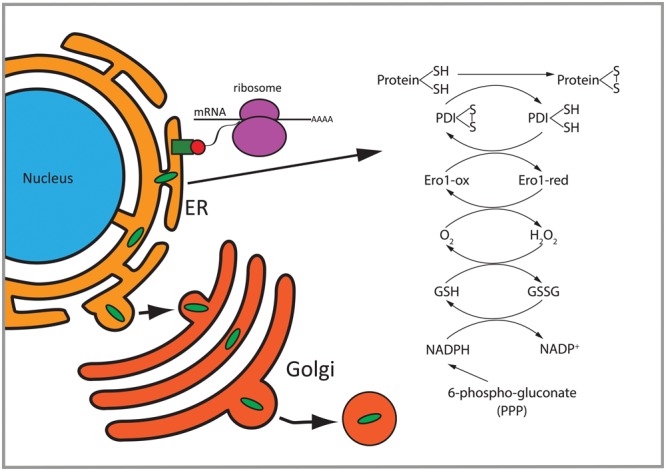
Overview of differentially regulated functions in *2b5^ho^* astrocytes. Proteins with a signal peptide or transmembrane domain enter the ER to be folded. Proteins going through the secretory pathway that are subjected to *N*-glycosylation and disulfide bond formation are enriched in the set of eIF2B𝜀^Arg191His^-regulated proteins. Disulfide bridge formation through oxidation of these proteins can induce H_2_O_2_. 6-Phospho-gluconate is involved in the production of NADPH which can be used to remove H_2_O_2_. PDI, protein disulfide isomerase; GSH, reduced glutathione; GSSG, oxidized glutathione; Ero1-ox, oxidized Ero1; Ero1-red, reduced Ero1; PPP, pentose phosphate pathway.

Could there be a link between the two main observations, i.e., the deregulated transport of some proteins in the ER and the increased 6P-gluconate concentrations? We think this is likely; although PPP is cytosolic, there are links to the ER. Part of the pathway appears to be associated with the ER (Stockwell et al., 2012) and NADPH produced by the PPP might be needed to combat ROS formation, which can be produced in the ER by processes related to oxidative protein folding. On the basis of the combined proteomic and metabolomic findings, we hypothesize that *2b5^ho^* astrocytes may accumulate ROS as a result of an increased flux of proteins (e.g., requiring correct disulfide bond formation) through the secretory pathway (**Figures 2, 8**). Correct disulfide bond formation is dependent on protein disulfide isomerases (PDIs) and Ero1 in the ER (Bechtel and Weerapana, 2017). This process can result in H_2_O_2_ as by-product and increased PPP flux might compensate by producing NADPH (Espinosa-Diez et al., 2015). 6P-gluconate is the only metabolite in the metabolic screen that was changed in *2b5^ho^* astrocytes and it is not completely clear whether this reflects increased production or decreased breakdown or a combination of both. There are indications that G6PDH is the only NADPH-producing enzyme activated upon oxidative stress and therefore it has been called “guardian of the cell redox potential” (Filosa et al., 2003). Lack of consistent changes over time in NADPH in *2b5^ho^* astrocytes suggest that the balance between H_2_O_2_ and NADPH in steady state is “successfully” compensated (**Figure 8**). On the basis of our results we cannot discriminate between a compensated system or a deregulated system, the latter leading to pathology (Bechtel and Weerapana, 2017).

## Conclusion

The 80 proteins differentially detected in the AHA-SILAC proteome are most likely translationally regulated as their mRNA levels are similar. Regulation thus must occur via increased synthesis or accumulation in *2b5^ho^* astrocytes. We observed that the function of some of these differentially affected proteins is linked to astrocyte differentiation, which is known to be disturbed in VWM. As we would expect from deregulated protein synthesis in *2b5^ho^* cells, the regulated proteins differ from their non-regulated counterparts in aspects of their 5′-UTRs. Altered synthesis may lead to an increase of proteins in the ER and secretory pathway without an overt effect on overall secretion. Possibly, increased demands of protein folding in the ER may result in generation of H_2_O_2_ or other ROS by-products (Espinosa-Diez et al., 2015). Thus, the increased synthesis of proteins that undergo disulfide formation and elevated 6P-gluconate might point to increased ROS in astrocytes, which may affect their function (**Figure 8**).

## Author Contributions

Substantial contributions to the conception or design of the work: LW, RP, CP, MvdK, and TA. The acquisition, analysis: LW, RP, EZ, CvB, TtB, and EP. Interpretation of data for the work: LW, RP, EZ, JK, CP, CB, MA, DS, and TA. Drafting the work or revising it critically for important intellectual content: LW, RP, EZ, CvB, TtB, EP, JK, CP, CB, MA, DS, MvdK, and TA. Final approval of the version to be published: LW, RP, EZ, CvB, TtB, EP, JK, CP, CB, MA, DS, MvdK, and TA. Agreement to be accountable for all aspects of the work in ensuring that questions related to the accuracy or integrity of any part of the work are appropriately investigated and resolved: LW, RP, EZ, CvB, TtB, EP, JK, CP, CB, MA, DS, MvdK, and TA.

## Conflict of Interest Statement

The authors declare that the research was conducted in the absence of any commercial or financial relationships that could be construed as a potential conflict of interest.

## Footnotes

1 http://unafold.rna.albany.edu//?q=DINAMelt/Quickfold

2 http://ron.cimr.cam.ac.uk/protocols/NucCyto.html
