# Supplementary material for: Proteomic and Metabolomic Analyses of Vanishing White Matter Mouse Astrocytes Reveal Deregulation of ER Functions
Source: Front Cell Neurosci. 2017 Dec 20;11:411. doi: 10.3389/fncel.2017.00411 (PMC5770689; doi:10.3389/fncel.2017.00411)
Supplement: Supplementary file 3 [file Image_1.PDF]

Supplementary Material

Proteomic and metabolomic analyses of vanishing white matter mouse astrocytes reveal deregulation of ER functions

Lisanne E. Wisse[1, +], Renske Penning[2, +], Esther A. Zaal[2], Carola G. M. van Berkel[1], Timo J. ter Braak[1], Emiel Polder[1], Justin W. Kenney[3 ,#a], Christopher G. Proud[3,#b], Celia R. Berkers[2], A. F. Maarten Altelaar[2], Dave Speijer[4], Marjo S. van der Knaap[1], Truus E.M. Abbink[1*].

+: contributed equally

***Correspondence:** Truus E.M. Abbink, g.abbink@vumc.nl

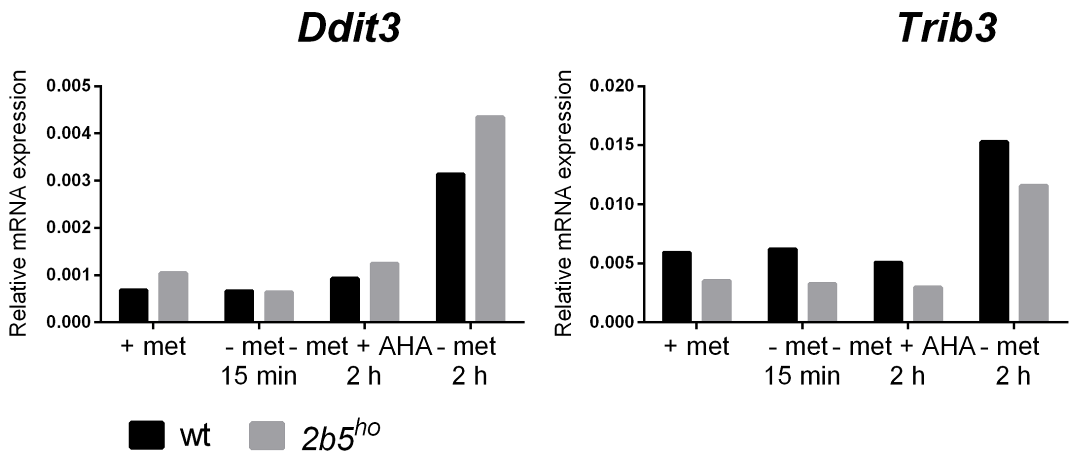

Supplementary Figure 1. Stress markers are not significantly induced in wt and *2b5^{ho}* astrocytes by AHA-labeling. AHA-labeling entails a 15-minute methionine starvation followed by a 2-hour AHA-labeling pulse, during which methionine in the culture medium is replaced with AHA. Cells were exposed to these assay conditions. A 2-hour methionine starvation was included as positive control for activation of the integrated stress response. RNA was isolated from cells and subjected to qPCR. The relative expression of *Ddit3* and *Trib3* mRNA was assessed as markers for activation of the integrated stress response. The 2-hour AHA labeling treatment did not activate the integrated stress response (- met + AHA 2h) when compared to the normal conditions (+ met). The 15-minute methionine starvation (- met 15 min) did not either. The 2-hour starvation increased *Ddit3* and *Trib3* mRNA expression as expected (- met 2 h). *Gapdh* mRNA was used as reference (n=1).

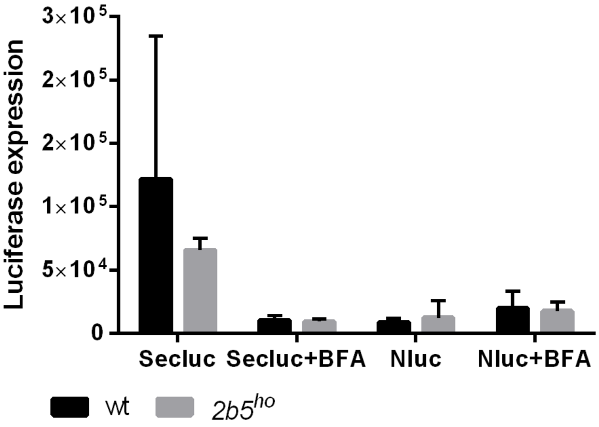

Supplementary Figure 2. Extracellular nanoluciferase activity after transfection of pNL1.3-*Gapdh* or pNL1.1-*Gapdh* in wt and *2b5*^{ho} astrocytes. Transfection of pNL1.3-*Gapdh* yields nanoluciferase with an *N*-terminal signal peptide (Secluc) that accumulates extracellularly. Transfection of pNL1.1-*Gapdh* yields nanoluciferase without this signal peptide (Nluc), which did not significantly accumulate extracellularly. Brefeldin A (BFA) inhibits the secretion of Secluc but not Nluc indicating that Secluc and not Nluc is secreted via the secretory pathway. The graph shows the mean ± SD. These findings indicate that the luminescence signal measured after pNL1.3-*Gapdh* transfection corresponds to actively secreted nanoluciferase and is not caused by nonspecific release of nanoluciferase.

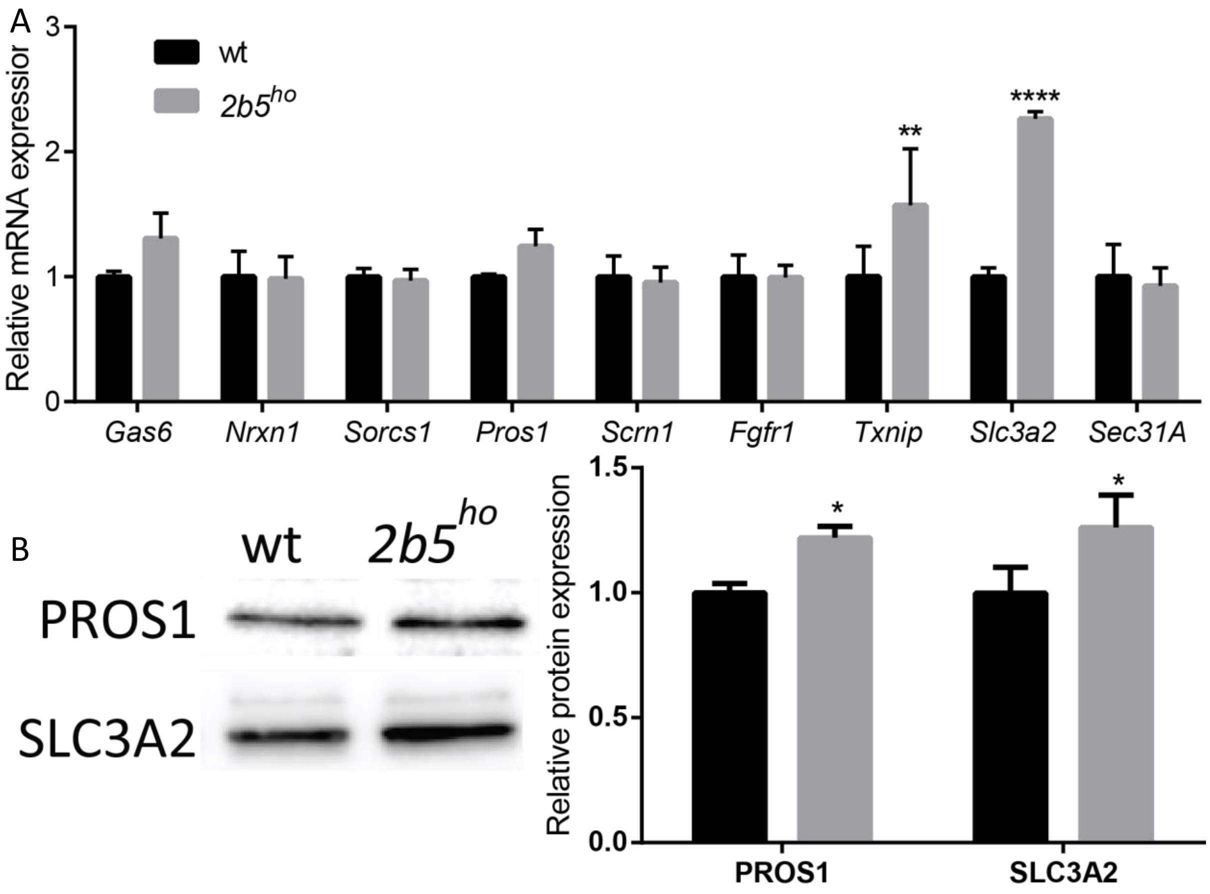

Supplementary Figure 3. mRNA and protein levels of the AHA-SILAC proteome in mouse brain. Panel A: candidate mRNA levels in brain samples (striatum) from 4 month-old mice. Most mRNAs are similar between wt and *2b5*^{ho} brain samples except *Txnip* and *Slc3a2*. Panel B: candidate protein levels in brain samples (striatum). Both candidate proteins, PROS1 and SLC3A2 are increased in *2b5*^{ho} mouse brain. Staining of all lanes including loading control are showed in supplementary figure 4. Graphs show average ± SD (n=3). *p<0.05, ** p<0.01, ****p<0.0001.

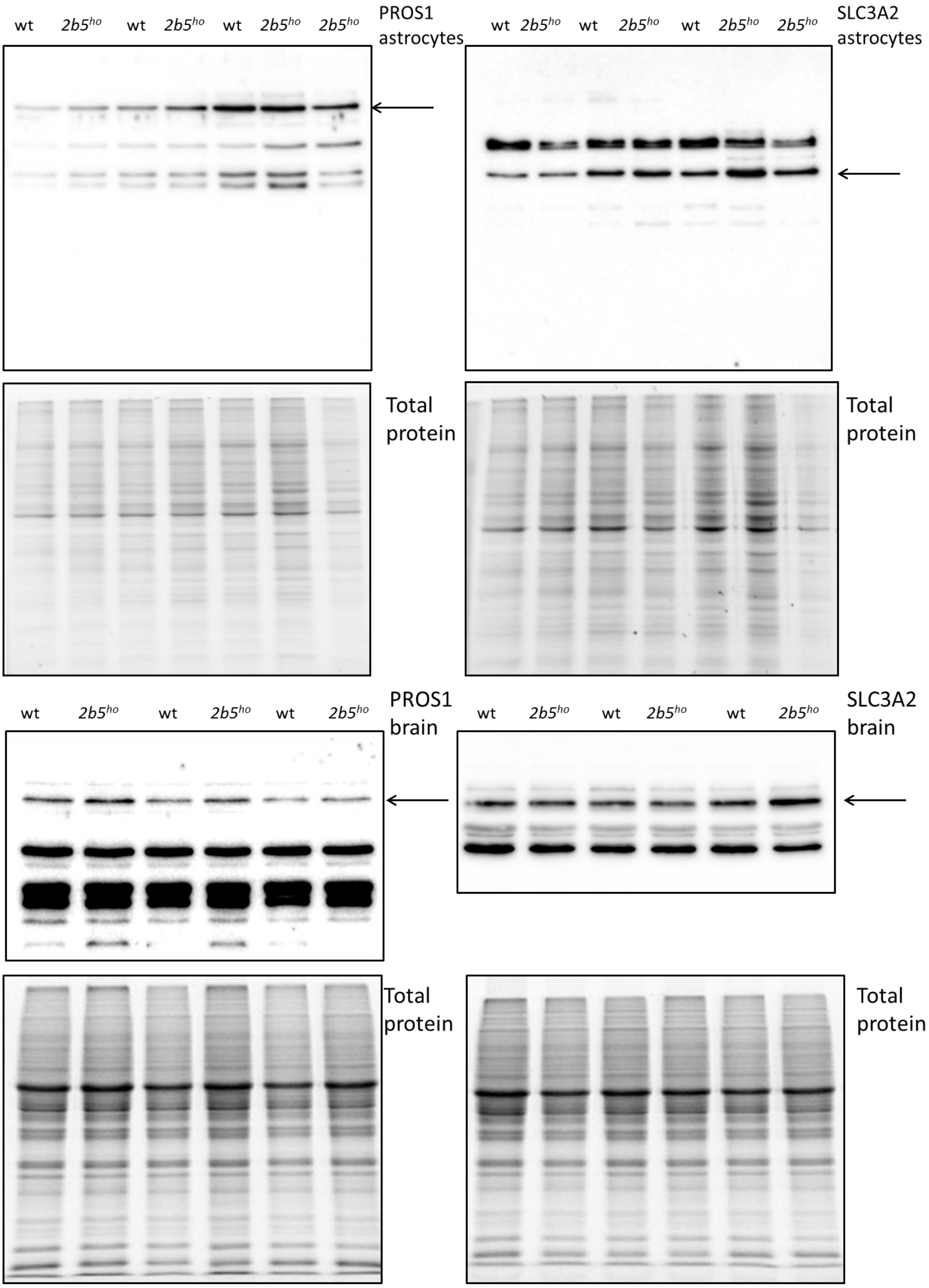

Supplementary Figure 4. Overview PROS1 and SLC3A2 western blots with loading controls. The top part shows the staining of PROS1 and SLC3a2 on astrocyte cultures with the total protein loading of the same blot underneath. The bottom part shows the staining of PROS1 and SLC3A2 on brain lysates with the total protein loading of the same blot underneath.

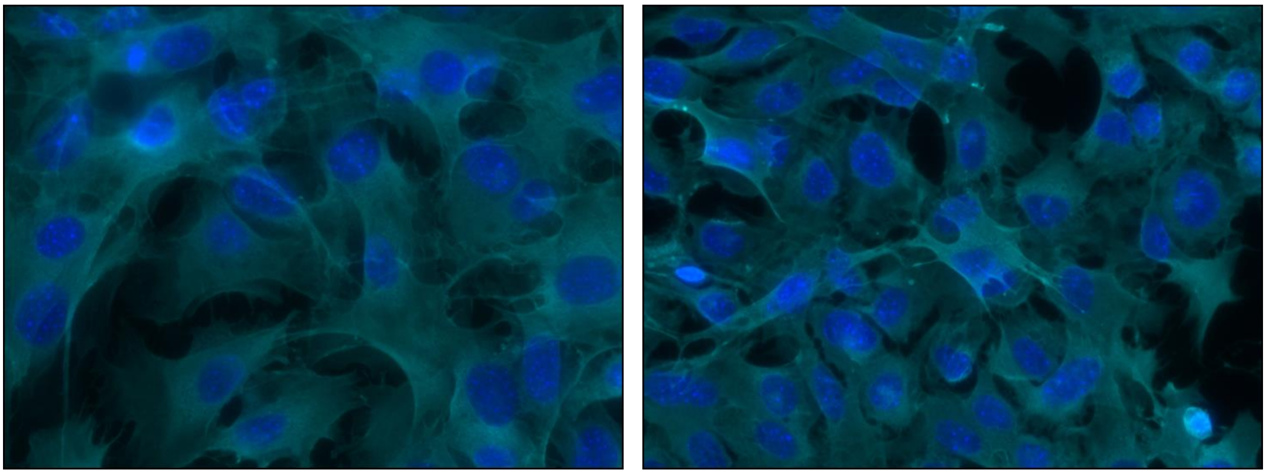

Supplementary Figure 5. Staining of AHA-labeled proteins demonstrates astrocytic morphology of wt and *2b5^{ho}* cells. Astrocyte cultures were treated with AHA for 2 h. AHA-labeled proteins were visualized using Click-iT™ Cell Reaction Buffer Kit (Invitrogen, C10269). The morphology of wt and *2b5^{ho}* astrocytes cultures demonstrates an astrocytic identity. Differences between wt and mutant astrocytes were not observed.
